# Hepatic granuloma mimicking recurrent lymphoma on ^18^F-FDG PET/CT in a patient with primary mediastinal diffuse large B-cell lymphoma

**DOI:** 10.22038/AOJNMB.2021.56876.1396

**Published:** 2022

**Authors:** Abdul Rahman Akkawi, Lynn Ezzeddine, Rita Chahinian, Firas Ershaid, Diala Merheb, Majd Mzeihem, Jean El-Cheikh, Mohamad Haidar

**Affiliations:** 1 Department of Clinical Diagnostic Radiology, American University of Beirut Medical Center, Beirut, Lebanon; 2 Department of Diagnostic Radiology, Saint George Hospital University Medical Center, University of Balamand, Beirut, Lebanon; 3 Department of Internal Medicine, Division of Hematology/Oncology, American University of Beirut Medical Center, Beirut, Lebanon; † These authors shared first authorship.

**Keywords:** F-18 PET CT, Large B cell lymphoma, Hepatic granuloma, Hepatic candidiasis

## Abstract

^18^F-Flurodeoxyglucose (FDG) PET/CT has been considered the modality of choice in detecting, staging, restaging and following-up with lymphoma patients. However, it has an uncertain role in differentiating hepatic lymphomatous relapse from other granulomatous diseases such as in candidiasis or sarcoidosis. Therefore, it is important to correlate the imaging findings with other modalities such as ultrasound, CT scan, MRI, and histology to direct the diagnosis and treatment. We present a case of a 33-year-old woman with large B-cell lymphoma in complete remission following treatment presenting with neutropenic fever following her final cycle of chemotherapy. Ultrasound of the abdomen and enhanced CT scan of the abdomen and pelvis were negative. The FDG PET/CT scan showed multiple FDG-avid hypodense hepatic lesions that were suggestive either of lymphoproliferative involvement or nonmalignant process. However, MRI of the abdomen performed four days later was suggestive of an infectious process, rather than a lymphoproliferative disorder. A subsequent CT-guided biopsy of a hepatic lesion showed granulomatous inflammation, with no evidence of malignancy or Tuberculosis. The patient was started on Caspofungin followed by Fluconazole. After 5 weeks, the clinical condition resolved, and the subsequent FDG PET/CT showed complete resolution of the FDG-avid multiple hepatic lesions.

## Introduction

 Despite the high accuracy of FDG PET/CT as an imaging method in following up of oncologic patients, it remains susceptible to pitfalls and artefacts limiting its specificity when differen-tiating between hepatic lymphoma, granuloma or infection (1-4). Some case reports have shown granulomatous diseases to mimic primary hepatic lymphoma (on imaging. (5-7). 

 Some reports have shown hepatosplenic candidiasis on FDG PET/CT in leukemia patient (7-8).

Herein is the case of a young patient with large B cells lymphoma in remission who was found to have a histologically proven liver granuloma of non-infectious etiology, mimicking a recurrent hepatic lymphoma which has resolved following a short period.

## Case Report

 This is the case of a 33-year-old female patient with a history of transverse myelitis on steroids, who presented to the hospital with a mediastinal mass. CT scan of the chest revealed supra-diaphragmatic lymphadenopathy, forming a mass at the anterior mediastinum, with direct bone invasion of sternum. A biopsy of the mass was performed and showed large B-cell lymphoma with features consistent with a primary thymic large B- cell lymphoma. As per the oncology team, the patient was subsequently started on Rituximab/Doxorubicin/ Cyclophos-phamide/Vincristine /Bleomycin/ Prednisone (R-ACVBP) chemotherapy regimen every 14 days. The first follow up FDG PET/CT showed complete remission after 2 cycles of chemo-therapy.

**Figure 1:**
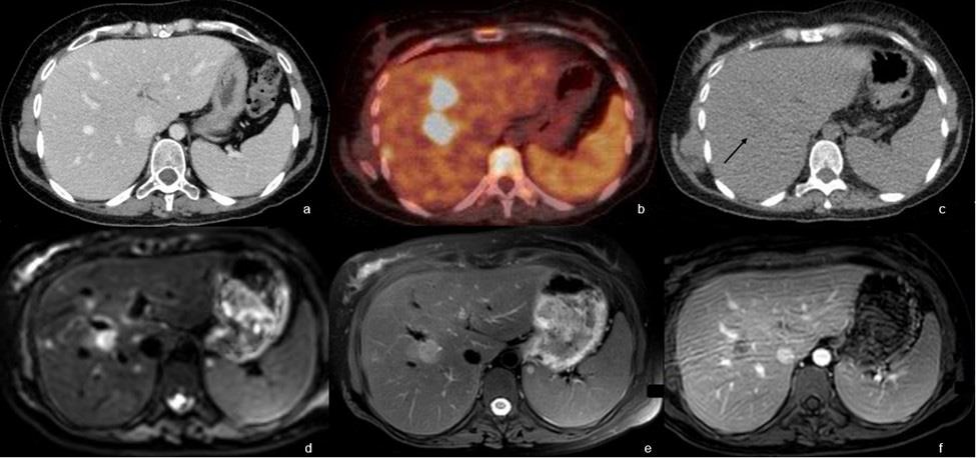
Axial enhanced CT scan of the abdomen shows no abnormality in the abdomen (**a**). Axial PET/CT fusion image of the abdomen shows few FDG-avid liver lesions with SUVmax 24 (**b**). Non-enhanced CT scan shows subtle hypodense lesions in the liver (black arrow) (**c**). Ill-defined lesion in segment VIII showing restriction of diffusion on DWI (**d**), on T2WI (**e**) and showing subtle rim enhancement and peri-lesion enhancement on T1WI post gadolinium administration (**f**)

 The patient presented to the hospital for neutropenic fever following her fourth chemotherapy cycle. Physical examination revealed fever and malaise with no other pertinent symptoms. Non-enhanced CT scan of the chest followed by a CT scan of the abdomen and pelvis with intravenous contrast administration were negative for any infectious process, mass or acute abnormality (Figure 1a). ^18^F-FDG performed four days later revealed multiple scattered hypodense intensely FDG-avid hepatic lesions suspicious of disease involvement with borderline enlarged spleen with mild diffuse increase. 

 FDG uptake was also identified (Figure 1b, 1c, 2a). Multiplanar multi-sequential MRI was performed for better characterization of the hepatic lesions, where it showed multiple ill-defined hepatic lesions in both lobes, mildly hyper-intense on T2WI, iso- to hypo-intense on T1WI with restriction of diffusion on diffusion weighted images (DWI) with a rim of perilesional enhancement (Figure 1d-1f). Findings were more suggestive of infectious process rather than lymphoproliferative disorder, warranting further evaluation by biopsy. Taking into consideration the risk of the immunosuppression status of our patient and the persistent fever, resistant to multiple lines of antibiotics, and the suggestive images on the PT Ct scan, a diagnosis of possible hepatosplenic candidiasis was made. For all these reasons, a treatment with IV caspofungin was started empirically with fever resolution associated with a net improvement of the clinical status of the patients. The patient was then shifted to high dose fluconazole with repeated PET scan on 30/03/2020 showing resolving lesions. Patient was later shifted on fluconazole 150 daily.

**Figure 2:**
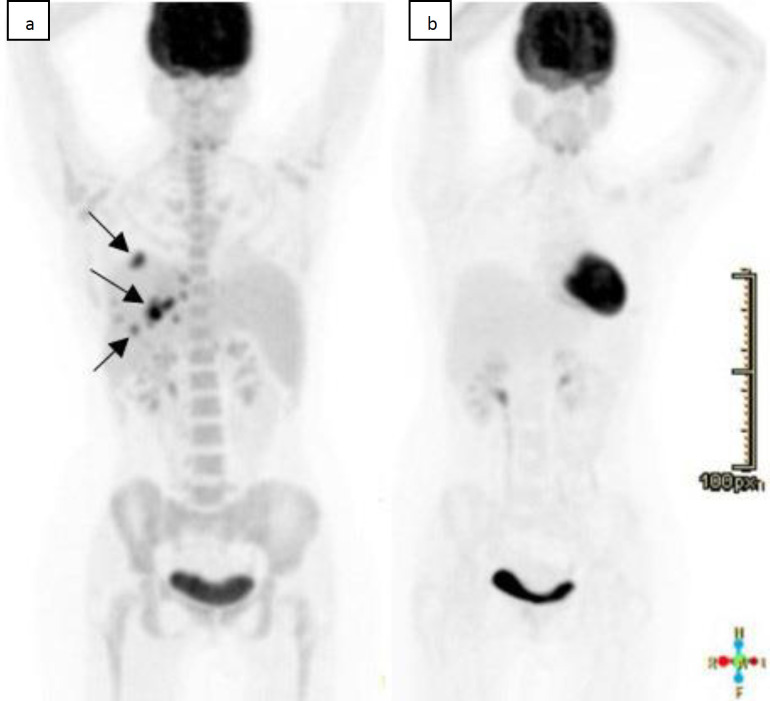
Maximum intensity projection (MIP) FDG-PET images. At the initial neutropenic stage (**a.**left) showing several FDG-avid liver lesions (arrows). Complete resolution of radiotracer uptake few weeks following treatment (**b.**right)

**Figure 3:**
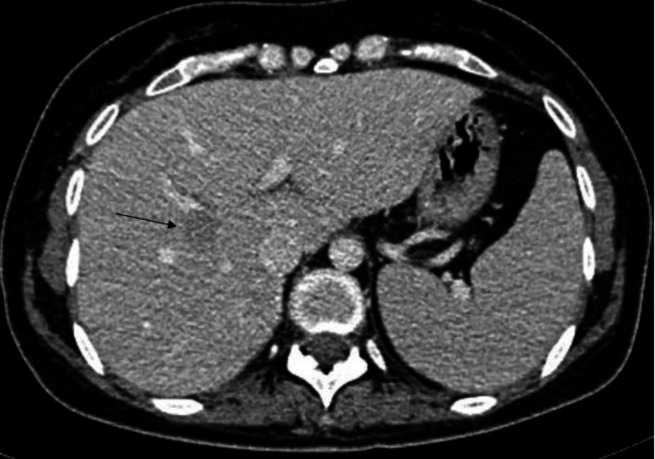
Axial CT image of the abdomen in late arterial phase for a CT-guided core biopsy denoting the hypodense lesion of segment VIII to be biopsied (arrow). To note intravenous contrast was administered prior to the CT-guided biopsy for better visualization of the previously described hepatic lesions under investigation

**Figure 4:**
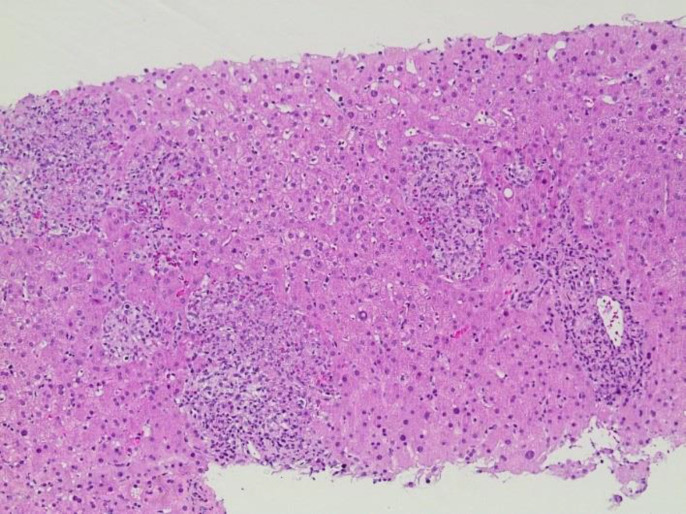
Histologic examination of the Core biopsy (H&E) shows multiple noncaseating granulomas involving hepatic lobules. On the right, there is a portal tract with chronic inflammation

 A CT-guided core biopsy was performed for the most FDG-avid lesion (Figure 3). The biopsy showed noncaseating granulomatous inflamm-ation that was negative for acid fast stain, GMS (Tb and pneumocystis carinii) and malignancy (Figure 4). Patient was therefore placed on on Fluconazole, Valtrex and Tavanic as per the oncology team recommendations. Repeat FDG PET/CT after 5 weeks for continued surveillance of disease progression revealed complete resolution of the previously demonstrated multiple FDG-avid liver lesions and splenic uptake.

## Discussion

 FDG PET/CT scan is a crucial tool in the diagnosis and follow up of oncologic patient, particularly in patient with lymphoma as it has higher sensitivity than conventional techniques (9). ^18^F-FDG, a glucose analog, allows for the evaluation of glucose metabolism in cells with elevated glucose requirements such as in malignancies and inflammatory diseases (9). It is notable to aggregate in inflammatory cells like lymphocytes, neutrophils, and macrophages in

different infectious or inflammatory conditions because of raised glucose conditions (3).

 In table 1, we describe the different pitfalls of high FDG uptake in hepatic lesions. Several articles have reported increased FDG uptake in the liver due to infections, benign and malignant tumors, and granulomatous diseases such as Candidiasis, Sarcoidosis, Histiocytosis, and Cryptococcosis (1-6). Hence the FDG avidity in granulomatous tissues can mimic malignancy such as lymphomas (4, 10, 11).

**Table 1:** Pitfalls of high FDG uptake in hepatic lesions

Granulomatous diseases:
Histiocytosis
Sarcoidosis
Cryptococcosis
Candidiasis
Infectious diseases:
Abscesses
Gallbladder inflammation
Benign Tumors:
Hepatic adenoma
Hemangioendothelioma
Focal Nodular Hyperplasia
Malignant Tumors:
Hepatocellular carcinoma
Gallbladder carcinoma
Metastasis
Lymphoma
Cholangiocarcinoma

 Ozer et al. reported two lymphoma patient who have developed sarcoidosis during their course of disease, which could have been misinterpreted as a lymphoma relapse on PET-CT due to their enhanced FDG uptake in mediastinal lymph nodes (12). It is thought that sarcoidosis development in oncology patients is due to an immunological reaction against tumor antigens, which can be mistaken for a lymphoma relapse (13). Therefore, it is crucial to highlight this correlation with other imaging tools. In 2014, London et al. reviewed 14 cases of sarcoidosis occurring after Lymphoma (13).

 Diffuse large B-cell lymphoma (DLBCL) is the most common aggressive lymphoma accounting for 25% of all non-Hodgkin lymphoma (5). Apart from the lymph nodes, spleen and bone marrow, the liver is the most common affected organ (5). However, data on the clinical significance and prevalence of focal liver lesions occurring in lymphoma patient with remission remain limited (13).

 On the other hand, hepatic candidiasis is a manifestation of dispersed candidiasis in immunocompromised patients which could mimic hepatic lymphoma on histological and functional imaging scu has CT scan, FDG-PET and MRI (14-16).

 The increased FDG uptake in the hepatic lesions of such patients, undergoing continuous treatment, may mimic the imaging characteristics of malignant involvement of the liver, which leads to false results of DLBCL relapse as seen in our case (1). In such cases, Van Prehn et al have suggested empirical treatment with Fluconazole in neutropenic patients with liver lesions showing no typical features for lymphomatous involvement on either imaging or biopsy, before planning additional surgery or chemotherapy (16).

 The distinction between recurrent lymphoma and granulomatous disease based on FDG PET/CT avidity remains limited with no distinctive features found neither in the former nor the latter. Therefore, prompt correlation and careful interpretation of additional imaging modalities are essential for the diagnosis of hepatic lesions in such cases. Nevertheless, a definitive diagnosis can be established through biopsy and histopathologic tissue sampling whenever feasible.

## Conclusion

 Abnormal liver uptake in ^18^F-FDG PET/CT scan in oncologic patient with lymphoma should be approached with attentiveness and awareness of its pitfalls, as different pattern of hepatic uptake has been described with no specific or pathognomonic features to any underlying etiology. Correlation with other imaging modalities and clinical findings remain crucial in the interpretation of the hepatic lesions and its altered activity. A sine qua non for the diagnosis of the hepatic lesion is by the biopsy and histopathologic sampling (1, 2, 17).
